# Supplementary figures and images for: Identification of a Potential Founder Effect of a Novel PDZD7 Variant Involved in Moderate-to-Severe Sensorineural Hearing Loss in Koreans
Source: Int J Mol Sci. 2019 Aug 26;20(17):4174. doi: 10.3390/ijms20174174 (PMC6747409; doi:10.3390/ijms20174174)

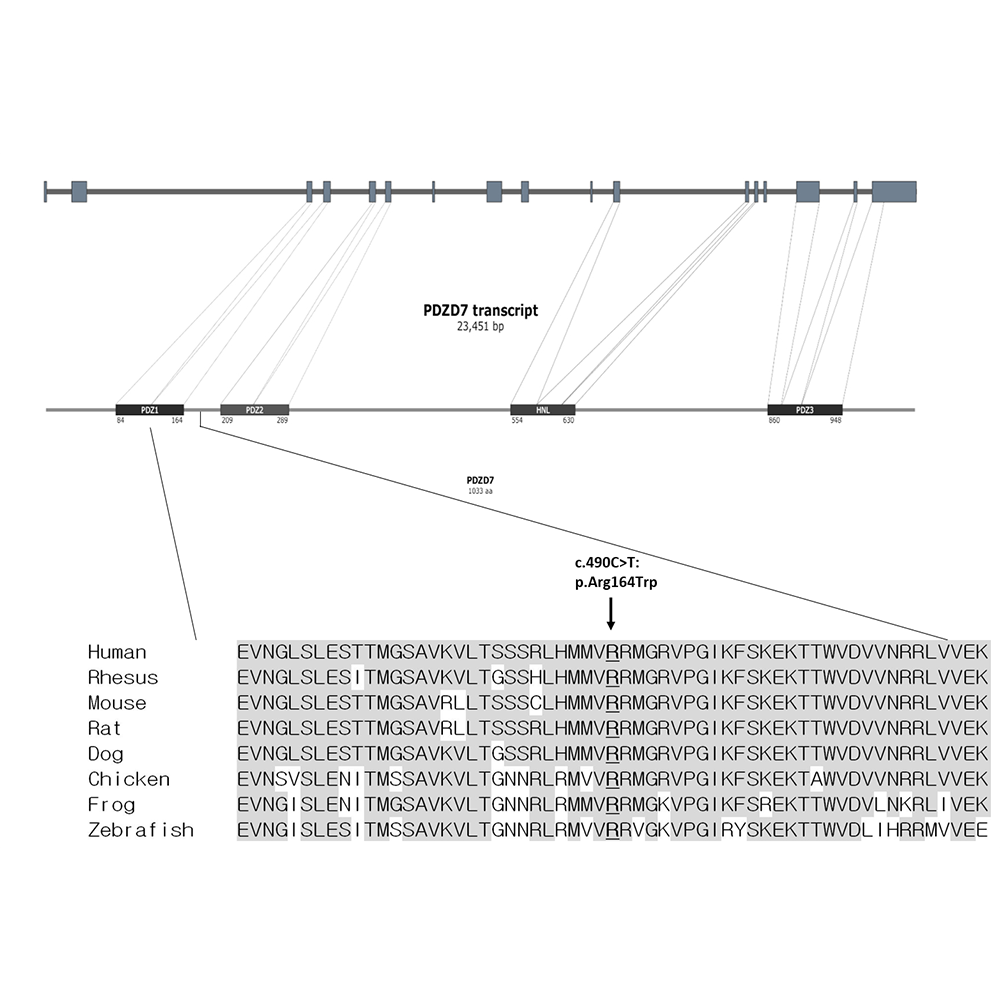

Supplement: Supplementary file 1 [file ijms-20-04174-s001.zip › ijms-571679-supplementary.tif]
